# Retraction Note: Regulation of TRIM24 by miR-511 modulates cell proliferation in gastric cancer

**DOI:** 10.1186/s13046-026-03838-9

**Published:** 2026-10-01

**Authors:** Ling Zhang, Quan Liao, Yi Wang, Feng Yu, Miao Feng, Xiaojun Xiang, Jianping Xiong

**Affiliations:** Department of Oncology, the First Affiliated Hospital of Nanchang University, 17 Yongwaizheng Street, Donghu District, Nanchang, 330006 Jiangxi Province China

**Retraction Note: J Exp Clin Cancer Res 36, 17 (2017)**

**https://doi.org/10.1186/s13046-017-0489-1**

The Editor-in-Chief has retracted this article.

After publication, concerns were raised regarding the data presented. The miRNA results were erroneously reported as normalized to U6 snRNA levels using primers that in fact amplify the messenger RNA LSM5. As a result, the normalization procedure described is incorrect, and the conclusions based on these data cannot be considered reliable. Additionally, the BGC823, MGC803 and SGC7901 cell lines used in this study have been reported to be contaminated with HeLa cervical cancer cells, making them unsuitable to use as models of gastric cancer. The Editor-in-Chief has therefore lost confidence in the data presented.

Ziling Fang, Jianping Xiong and Xiaojun Xiang agree with this retraction. Remaining authors have not responded to correspondence regarding this retraction.

